# Palm Oil Fuel Ash-Based Eco-Friendly Concrete Composite: A Critical Review of the Long-Term Properties

**DOI:** 10.3390/ma14227074

**Published:** 2021-11-22

**Authors:** Mugahed Amran, Yeong Huei Lee, Roman Fediuk, Gunasekaran Murali, Mohammad Ali Mosaberpanah, Togay Ozbakkaloglu, Yee Yong Lee, Nikolai Vatin, Sergey Klyuev, Maria Karelia

**Affiliations:** 1 Department of Civil Engineering, College of Engineering, Prince Sattam Bin Abdulaziz University, Alkharj 16273, Saudi Arabia; 2 Department of Civil Engineering, Faculty of Engineering and IT, Amran University, Quhal 9677, Yemen; 3 Department of Civil and Construction Engineering, Faculty of Engineering and Science, Curtin University, CDT 250, Miri 98009, Malaysia; yhlee@civil.my; 4 Polytechnic Institute, Far Eastern Federal University, 690922 Vladivostok, Russia; roman44@yandex.ru; 5 School of Civil Engineering, SASTRA Deemed to Be University, Thanjavur 613404, India; murali@civil.sastra.ac.in; 6 Department of Civil Engineering, Cyprus International University, 99258 Nicosia, Turkey; mmosaberpanah@ciu.edu.tr; 7 Ingram School of Engineering, Texas State University, San Marcos, TX 78666, USA; togay.oz@txstate.edu; 8 Department of Civil Engineering, Faculty of Engineering, Universiti Malaysia Sarawak, Kota Samarahan 94300, Malaysia; yylee@unimas.my; 9 Peter the Great St. Petersburg Polytechnic University, 195251 St. Petersburg, Russia; vatin@mail.ru; 10 Department of Theoretical Mechanics and Strength of Materials, Belgorod State Technological University Named after V.G. Shukhov, 308012 Belgorod, Russia; klyuyev@yandex.ru; 11 Department of Machinery Parts and Theory of Mechanisms, Moscow Automobile and Road Construction University, 125319 Moscow, Russia; karelinamu@mail.ru

**Keywords:** palm oil fuel ash, long-term properties, geopolymer concrete, durability

## Abstract

Rapid global infrastructural developments and advanced material science, amongst other factors, have escalated the demand for concrete. Cement, which is an integral part of concrete, binds the various individual solid materials to form a cohesive mass. Its production to a large extent emits many tons of greenhouse gases, with nearly 10% of global carbon (IV) oxide (CO_2_) emanating from cement production. This, coupled with an increase in the advocacy for environmental sustainability, has led to the development of various innovative solutions and supplementary cementitious materials. These aims to substantially reduce the overall volume of cement required in concrete and to meet the consistently increasing demand for concrete, which is projected to increase as a result of rapid construction and infrastructural development trends. Palm oil fuel ash (POFA), an industrial byproduct that is a result of the incineration of palm oil wastes due to electrical generation in power plants has unique properties, as it is a very reactive materials with robust pozzolanic tendencies, and which exhibits adequate micro-filling capabilities. In this study, a review on the material sources, affecting factors, and durability characteristics of POFA are carefully appraised. Moreover, in this study, a review of correlated literature with a broad spectrum of insights into the likely utilization of POFA-based eco-friendly concrete composites as a green material for the present construction of modern buildings is presented.

## 1. Introduction

Global warming, climate change, and more recently the concept of environmental sustainability have been major concerns as their adverse effects cannot be over-emphasized [1]. The introduction and incessant accumulation of harmful waste materials into the environment, along with certain industrial processes, which emit excessive volumes of greenhouse gases as by-products, tend to sabotage efforts to achieve a cleaner environment [2]. In particular, the construction industry, with its consistent upsurge in urbanization, as well as population growth, is vital for rapid global infrastructural development [3]. However, the gross demand for cement, an integral constituent of concrete utilized in construction works, and other allied materials continues to grow at an exponential rate. Cement production is usually characterized by a large consumption of natural resources and energy, and is accompanied by the emission of greenhouse gases [1,4]. It is aptly considered as one of the most globally utilized commodities [5]. Likewise, it is worth noting that producing 1 ton of cement results in approximately 1 ton of CO_2_ emissions [6]. Apparently, 7–10% of global anthropogenic CO_2_ emissions is linked to cement production; a careful comparison with the aviation industry, which produces 2.8% CO_2_ emissions, shows three times lower volume of carbon (IV) oxide emissions [7,8]. If the exponential demand for cement must be met, and the need for ecologically sustainable systems achieved, alternative materials need to be explored [9].

The use of supplementary cementitious materials (SCMs) has continued to gain increased attention [10,11,12,13,14]. There are a number of silica-containing SCMs of both natural and man-made origins, and it is especially important to use the waste from various industries as these materials. Natural SCMs include opoka (86–92% SiO_2_), trepel (85–89%), volcanic tuff (9–23%), and diatomite (82–87%). Technogenic SCMs include silica fume (over 90%), metakaolin (53–54%), rice husk ash (90–99%), fly ash (60–70%), ash and slag mix (50–60%), granulated blast furnace slag (28–38%), and red mud (3–50%) [15,16,17,18,19,20,21].

**Figure 1:**
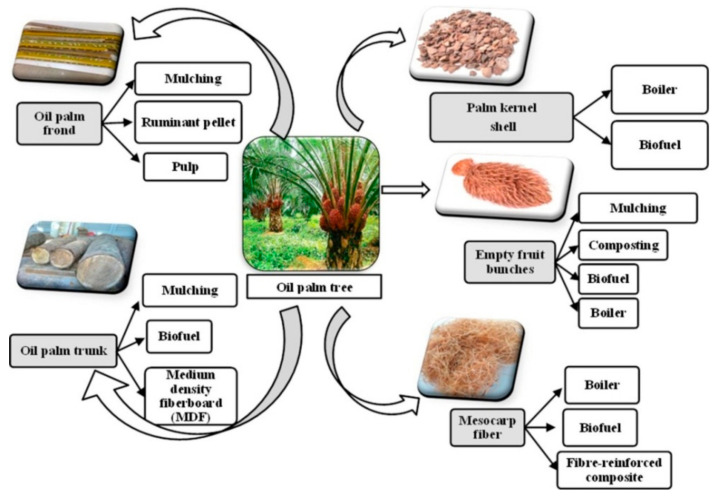
The present use of oil palm solid wastes [22]. Reprinted with permission from Elsevier [22].

Palm oil fuel ash (POFA) has been deemed a potential alternative from agricultural waste (as seen in Figure 1 [22]), it is an industrial by-product which is a resultant of the incineration of palm oil wastes in oil mills; it has unique properties and is a very reactive material with robust pozzolanic tendencies. As shown in Figure 1, the utilization of oil palm solid wastes has been classified into five main categories: oil pam frond, oil palm trunk, mesocarp fiber, empty fruit bunches, and palm kernel shell. Each part of palm solid wastes can be utilized for different uses. In regards to classification, the tall-stemmed oil palm tree is from the Palmea family. Several countries cultivate this tree, among which Zaire, Nigeria, Malaysia, Indonesia, Ecuador, Colombia, and the Benin Republic, as well as Malaysia, are dominant in the production of palm oil-related products [23]. Reports from Malaysia estimate that, annually, the total solid waste produced by over two hundred oil mills averages about ten million tons [24]; subsequently, the by-products are used as alternative cheap sources of fuel to fire up palm oil mill boilers, resulting in the generation of a large volume of ash [25].

With the millions of tons of ash generated per year in the Far East, adequate disposal has become a major challenge, and putting it to use in more profitable ways has become a priority [26].

The waste materials from the palm oil industry, such as shells, fiber, empty fruit bunches, and kernels, are used to produce energy in power plants through burning them [27] and result in POFA as a byproduct [28,29]. Due to its cementitious properties, POFA can be used as a partial replacement for cement in a concrete matrix [30]. Parallel to demand, the production of palm oil continues to increase and raises environmental issues due to its massive amount of waste materials. As one of the largest palm oil exporters, Malaysia is able to produce approximately 10 million tons of POFA per year [31,32,33], whereas, 104 million tons of POFA are being produced in Thailand, and this is expected to grow as a function of time [27].

Moving sustainability forward, applying industrial and agricultural wastes in concrete materials has shown increasing trends and interest [30,34,35]. Landfills are where waste materials will disposed of forever [36] and by the increasing amount of waste materials, they are eventually dumped into the environment and cover a larger area of these landfills (e.g., municipal solid waste landfills, industrial waste landfills, hazardous waste landfills, and green waste landfills), which cause several environmental pollution problems. The mining of landfills will create a great benefit in terms of addressing both environmental and economic issues [12]. Agricultural wastes possess better properties than cement in concrete, as cement production generates a higher carbon footprint [35]. It can be proven that POFA consumes less energy during production [37,38]. In Malaysia, there was an excess of 1000 tons of POFA dumped into landfills without exploring its second life [39]. The application of palm oil fuel ash as a cement replacing material is able to reduce concrete production costs and also proffers better solid waste management towards a cleaner environment.

POFA is categorized as an ash material and is obtained by burning palm oil waste materials, primarily kernel shell and husk [32]. Excessive POFA is dumped in landfills as usual practice, making it increasingly difficult in the context of proper land usage [40]. To minimize potential risks to the environment, it is necessary to explore its usage in other industries. Researchers started investigations into the possibility of implementing POFA as partial cement replacement for concrete in the 1990s [41], when a strength reduction of 20 to 50% was found with a POFA cement replacement of 10 to 50%. From the cement replacement, it was found that POFA was able to reduce sulfate attack [39]. Regarding pozzolanic reaction, several studies have been conducted with different POFA finenesses [42], using a constant water–binder ratio of 0.5, ground river sand (GRS), and ground POFA (GPOFA) to replace ordinary Portland cement by 10 to 40% by weight of cementitious constituents, and the results revealed that the higher fineness may have been beneficial for the concrete properties.

This paper aims to present a comprehensive review on the reuse and utilization of POFA as a by-product from the palm oil industry, which is achieved by incineration of sufficient amounts of waste materials, such as the husk, fiber, and shell of palm oil plants and its effect as supplementary cementitious materials (SCMs) on the mechanical and durability properties of eco-friendly concrete composites.

## 2. Source of Byproduct POFA

**Figure 2:**
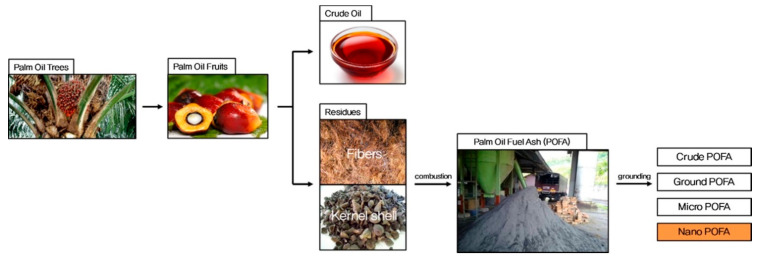
Production procedure of POFA [52]. Reprinted with permission from Elsevier [52].

POFA is a by-product from the palm oil industry, which is achieved through the incineration of sufficient amounts of waste materials, such as the husk, fiber, and shell of palm oil plants [23] (shown in Figure 2). Burning of these wastes generate energy at palm oil mills and achieves high temperatures of 1000 °C [43]; they are also often referred to as fuel [44] and results in about 5% of the total waste weight of POFA [28,43]. Annually, more than 3 million tons [43] and more than 100,000 tons [30,45] of POFA are produced in Malaysia and Thailand, respectively. With consideration of environmental issues and overall costs, energy generation through waste materials from palm oil mills for the purpose of heating boilers is a general solution in the industry. The produced palm oil is about 25% of the total weight of the raw material, while about 75% is dry waste biomass [27,46]. For the total palm oil waste, 75% consists of trunks and fronds and are recycled and applied as plant fertilizer, the remaining 25% containing mesocarp fiber, void fruit bunches, and hard palm kernel shells are channeled for generating energy or electricity for the production line [47,48]. There are several methods to prepare POFA: (1) 24 h of moisture removal by drying at 105 ± 5 °C [43]; (2) through combustion at up to 1000 °C and passing samples through a 1.18-mm sieve [30]; and (3) a three-step preparation of ultrafine POFA [49]. Moisture and coarse particle removal are the first steps in an oven at 105 ± 5 °C and the substances are passed through a 300-mm sieve to remove particles that failed to burn. A high fineness of POFA is usually obtained through the second step, by grinding followed by high-temperature combustion at 500 ± 50 °C to remove unburned carbon. Similar to step 2, the third step requires further grinding to get finer POFA particles. POFA predominantly consists of non-crystalline silica and alumina, which makes it a potent SCM [50]. However, only a relatively small quantity of palm oil waste is utilized for POFA, the rest is disposed of in landfills, leading to severe environmental challenges [51].

## 3. Factors Affecting the Long-Term Properties of POFA-Based Concretes

### 3.1. Ratio of Replacement

**Figure 3:**
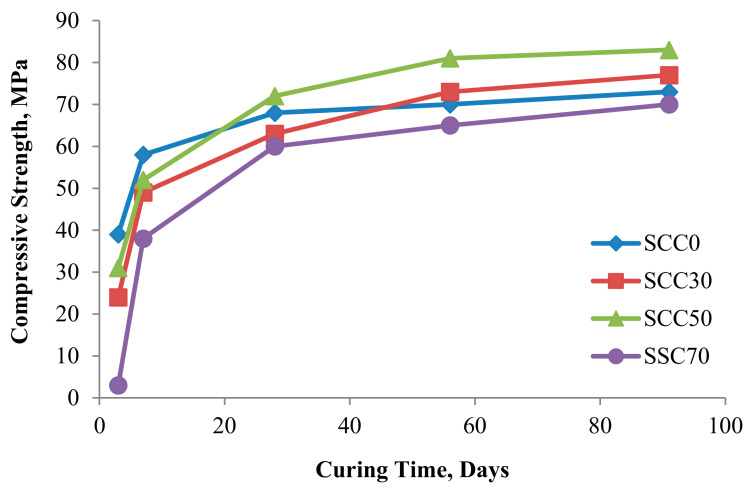
Development of SCC compressive strength under water curing [61]. Annotations: SCC contains 0% POFA, SCC30—30%, SCC50—50%, SCC70—70%. Reprinted with permission from Elsevier [61].

POFA, as an SCM, has a high carbon content, loss on ignition (LOI), and enormous particle size, making it inactive. Compared with normal POFA, ultrafine POFA (UPOFA) has better properties after extensive grinding [53]. This can be determined from its higher compressive strength [54], as it is used in high-strength concrete (HSC) and self-compacting concrete (SCC) [55]. In contrast, a high replacement level of POFA may reduce compressive strength and workability [56,57]. Regarding this problem, increasing its fineness and further heating to eliminate carbon content are recommended to improve its properties [56,58]. From [59], it can be seen that treated POFA (TPOFA) exhibited greater improved concrete performance than ground POFA (GPOFA) due to a reduction in LOI and enhanced chemical composition. UPOFA as a cement replacement has been studied using different particle sizes in the context of the performance of mechanical properties in cement mortar [60]; smaller sized particles tended to give enhanced compression strength for alkaline-activated mortar. Similarly, Hamada et al. [57] found that 30% UPOFA enhanced compression strength and workability in palm oil clinker concrete. UPOFA with particles size ranging between 100 and 150 nm have also been studied [52]. Results from TEM and X-ray diffraction (XRD) showed that nano-sized POFA had no effect on early strength generation as a result of high pozzolanic reaction and formation of calcium–silicate–hydrate (C–S–H) gels. Therefore, the reduction of early compression strength is usually compensated for by increasing the cement replacement level using TPOFA [61] (Figure 3). In addition, with an increase in curing time, a better mechanical performance were achieved with TPOFA concrete [49]; a strength range of about 90 MPa was attained for high-volume UPOFA concrete for a prolonged curing age. POFA has proven the ability of concrete to attain high strength values as concrete age increases. An optimum geo-polymer mortar 7-day compressive strength was obtained using 65% cement replacement with a value of 47.27 ± 5.0 MPa and was determined using statistical analyses [62]. Moreover, with UPOFA up to 80% cement replacement level, the compression strength value was recorded as 45 MPa after 360 curing days [63]. However, high-volume TPOFA replacement has been found to reduce SCC compressive strength at an early curing age [45], while it increases with a later concrete age. In general, POFA, as partial cement replacement, may reduce mechanical performance (compressive strength) at an early curing age due to the low pozzolanic activities in concrete matrix.

### 3.2. Effect of POFA as a SCM in Concrete

**Figure 4:**
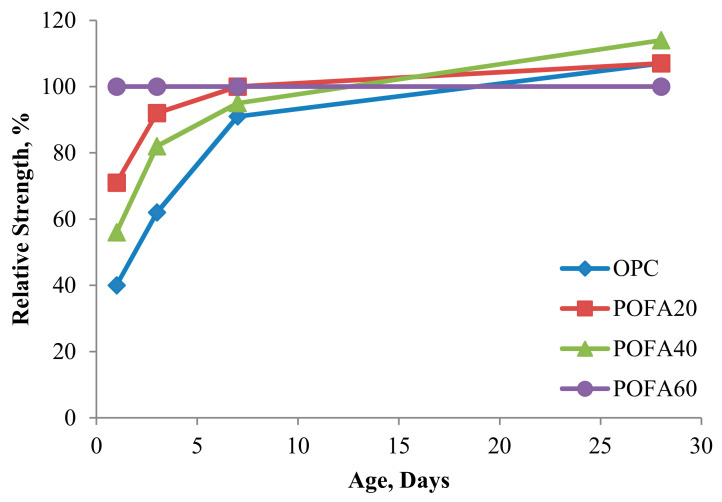
Effect of ultrafine-POFA on the development of compressive strength of high-strength green concrete [56]. Annotations: OPC contains 0% POFA, POFA20—20%, POFA40—40%, POFA60—60%. Reprinted with permission from Elsevier [56].

Enhancing concrete properties is the main reason that POFA is applied in concrete manufacturing [64]. For example, compressive strength, expansion due to magnesium sulfate attack, and initial and final setting time were investigated for POFA concrete, where concrete with POFA delayed the setting time was governed by POFA replacement level and degree of fineness [28]. The heat of POFA concrete was also studied and it was revealed that the hydration heat of concrete relies mainly on the form of chemical material interactions [32]. Improvements in compressive strength, thermal resistance, and flexural strength were found when introducing POFA into foamed concrete with a density of 1300 ± 50 kg/m^3^ [65]. The 10–15% POFA cement replacement in geo-polymer was found as to be the optimum level of replacement for a better compressive strength using geo-polymer concrete with a binder material and coarse aggregate, comprised of ground granulated blast-furnace slag (GGBS) and oil palm shell, respectively [66], and it was found contrast to the work in [67]. It was also found that 20% POFA cement replacement had a lower water permeability than OPC concrete [40]. As shown in Figure 4, previous studies [56,68] utilized an enormous volume (60%) of ultra-fine POFA to replace cement, which had significant silica content.

Durability is another concern for concrete structures, as materials gradually erode, reducing the serviceability period of infrastructure. Therefore, introducing FA and POFA into concrete have been investigated for durability and corrosion resistance [69]. Regarding the thermal properties of concrete, POFA concrete possessed a better resistance to aggressive environments [25]. In POFA concrete, the fineness can be increased using pozzolanic material, where more C–S–H gels formed from the interaction of Al_2_O_3_ and SiO_2_ with Ca(OH)_2,_ hence increasing the sulfate resistance and reducing Ca(OH)_2_ content [70].

Demonstrating the benefits of waste utilization from the oil palm industry in concrete production should be promoted for related agencies for further implementation [23]. It is concluded that POFA is recommended for application as a pozzolan in a concrete matrix to achieve a desired strength and durability in concrete production.

The interest in introducing SCMs into conventional concrete has increased the desire to produce better chemical and physical characteristics [71,72]. The physical properties, strength characteristics, and durability were studied for POFA as a SCM in cement paste [73]. POFA concrete research has been massively investigated in warm climate countries, where, due to the reduction in coal fly ash and the increasing trend in cement demand, new SCMs should be discovered for use as a cement replacement, even in cold climate countries [73,74]. High strength concrete has been studied in Thailand, with POFA as a SCM [30], and other research has also investigated the physical and chemical properties of both fresh and hardened states [75]; these states were highly resistant to chloride and carbonation and showed a lower permeability and shrinkage over long-term durations. As the palm oil industry is dominant in Southeast Asia [53], POFA, a by-product from palm oil mills, serves as the newest SCM addition to cementitious materials [32].

**Figure 5:**
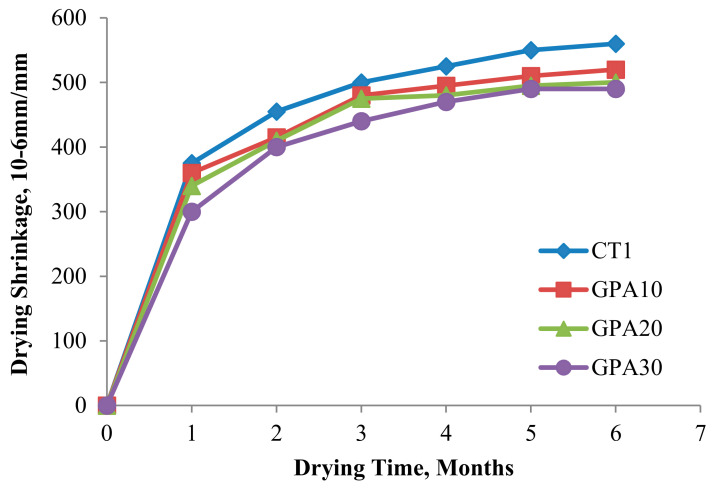
Effect of POFA as a SCM on drying shrinkage [30]. Annotations: GTI contains 0% POFA, GPA10—10%, GPA20—20%, GPA30—30%. Reprinted with permission from Elsevier [30].

Agro-based waste materials, such as rice husk ash (RHA), FA, and POFA as pozzolanic materials or SCM in concrete matrix have been studied [76]. By improving long-term concrete properties, SCMs, such as POFA, tend to benefit in terms of pore structures, with a higher resistance to sulfate and acid attacks and a reduction in concrete costs. However, a strength reduction may be found with excessive amounts of SCMs due to the low content of portlandite [77,78]. A concrete strength of 70 MPa at 90 days of curing has been obtained and permeability, drying shrinkage, and sulfate resistance were examined for POFA concrete [30] (Figure 5); a lower drying shrinkage and water permeability were discovered as the replacement level of GPOFA increased. Drying shrinkage of high strength POFA concrete was studied and is shown in Figure 5. Approximately 70% of the drying shrinkage was found in early stage (first 3 months) and similar trends were found in other studies [79,80]; the highest values were found at 6 months. POFA may be a potential cement replacement that is more economical and environmentally friendly. Its potential as a SCM may decrease if a higher carbon content is found in POFA particles [81].

### 3.3. POFA in SCC

**Figure 6:**
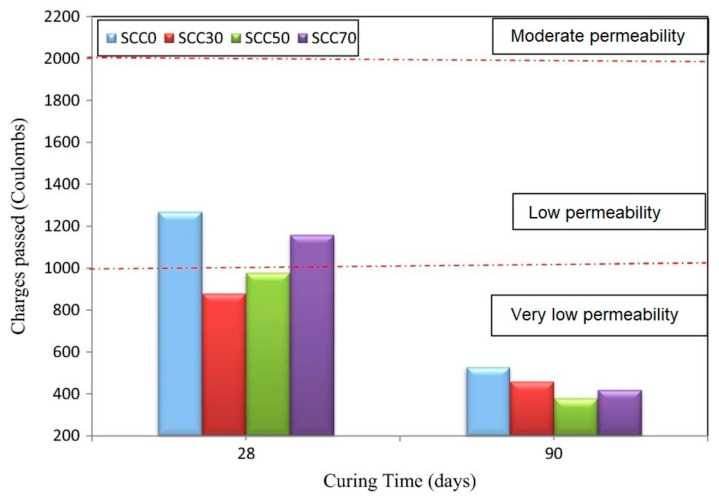
RCPT at 28 and 90 days [61]. Annotations: SCC contains 0% POFA, SCC30—30%, SCC50—50%, SCC70—70%. Reprinted with permission from Elsevier [61].

Another use of POFA is in producing self-compacting concrete for application in a narrow area of industry where compaction cannot be done [82], for instance, areas congested by massive reinforcements. SCC requires a smaller aggregate size without any strength reduction, which, in turn, implies additional costs. Partially, cement replacement using a SCM is an alternative to lowering the additional costs [83]. With this, a more sustainable concrete, SCC with POFA, can be enhanced and energy savings can be achieved [84]. A high replacement level of POFA with a considerably large amount of silica (up to 66%) can be used to produce various concrete types [85]. As illustrated in Figure 6 [61], rapid chloride permeability tests have been conducted and it was revealed that durability improved with curing age, and cement replacement with POFA where total SCC charges for 0, 30, 50, and 70 were 1270, 880, 980, and 1160 coulombs at day 28 and to 530, 460, 380, and 420 at day 90, respectively. All these investigated concretes are classified as low and very-low permeable concretes at a curing age of 28 and 90 days, with reference to ASTM 1202 [86]. Similar to concrete with modified treated POFA (MT-POFA), their enhanced resistance was ascribed to the additional physical coating produced during reaction with calcium hydroxide [61] (Figure 6). The effect of this reaction is to produce a denser microstructure with further production of additional C–S–H bonding, which reduces concrete porosity and permeability.

### 3.4. Effect the Degree of Fineness of POFA on the Blended Cement Paste

Fineness is deemed as one of the most notable characteristics of POFA. The rate of hydration and the nature of pozzolanic materials rely on the fineness of POFA particles, where a higher degree of fineness increases the compressive strength of concrete [43,87]. Grinding in ball mills is used to obtain finer particle sizes of POFA [28,30,54] and to reduce its porosity [88]. Passing or retaining using sieve no. 325 is a method to identify the fineness of POFA [89]. The specific area of OPC is relatively smaller than that of GPOFA due to particle size. Treatment inside palm oil mills may affect the chemical and physical properties of POFA [73]. Different POFAs with finenesses ranging from 300 to 1800 m^2^/kg may be obtained from different treatments, which include acidic or alkaline pretreatments, calcination temperature, and others [27]. Khalid et al. [87] claimed that a higher degree of fineness can be obtained through a grinding process using a rod bar. Furthermore, a superplasticizer may be absorbed by unburned carbon and affect concrete workability and this can be avoided by heating it up to 500 °C for one hour [56]. It was found that after a half an hour of grinding a 4930 cm^2^/g specific surface area was able to be achieved, and only 10.5% of the ash was found to be retained in a 45-µm sieve [53].

Higher degree of fineness can be obtained and a higher packing effect of POFA cement the is found when the paste increases with more fineness. A pozzolanic reaction between 12 to 23% with average particle sizes but lower than that of cement at a characteristic concrete age was achieved [42,45]. The high fineness of POFA was found to be able to replace type I Portland cement at a 30% replacement level. Durability could be improved by the packing effect and pozzolanic properties through pore refinement and reduction of Ca(OH)_2_ [26]. The reactivity with free-lime with extra silica content generated extra C–S–H gels and, thus, increased POFA concrete strength [53,90]. Furthermore, pozzolanic activity could be increased through the packing influence, where LOI content reduced the chemical structure, particularly that of SOi_2_, from 69.02% to 59.17% [45]. In addition, a high pozzolanic activity could be obtained through a decrease in Ca(OH)_2_, MgO, and SO_3_ [33,91,92,93,94].

In terms of microstructure property, as discussed before, additional C–S–H bonding resulted in a more dense concrete microstructure and also improved the interfacial bond, which improved the strength, transport characteristics, and durability of concrete [95,96] as a result of its micro-filling capability and pozzolanic reactivity. GPOFA was found to be an amorphous silica material [97]. Blended cement pastes with coarse POFA was found to have higher compressive strengths than OPC cement paste, as GPOFA contained a very fine POFA. The microstructures of high strength concretes (HSC) with POFA were investigated at curing ages of 3 and 7 days [23], and a porous morphology was observed at 3 days and ettringite crystals were observed at 7 days via SEM scanning. Some hexagonal platelets of Ca(OH)_2_ was also observed in several low strength samples at 7 days. In general, 20% cement replacement in HSC with POFA showed the highest compression strength value and the lowest permeable porosity [95].

### 3.5. Shape and Size of Particles

Generally, ground POFA is smaller than unground POFA in term of particle size. Ground POFA has angular and irregular shapes (crushed particles) and unground has a spherical shape with pores [54]. Ground POFA was found to have smaller particle sizes than cement, ranging from 7.2 to 10.1 µm, compared to bigger cement particles, which ranged between 10–20 µm [59]. Scanning electron microscopy (SEM) is a popular tool to investigate the microstructure of concrete [43,98,99,100,101,102]. Most of the POFA particles were sphere-shaped and had a lower specific gravity compared to cement particles [103]. Field emission scanning electron microscopy (FESEM) was utilized to analyze the morphological structures of UPOFA [104] and analysis revealed that the UPOFA particles were irregular and thinner. Similarly, with the found air gaps, POFA has irregular and thinner shapes that resulted from the crushed particles [105,106].

## 4. Durability Properties

Durability denotes the resistance of concrete to decay and disintegration. One of the main characteristics that influences durability is permeability, which can increase the rate of capillary absorption (RoCA) and the resistance of concrete to potentially deleterious materials. By ensuring a full compaction of admixtures and adequate curing, POFA concrete is sufficiently able to withstand environmental effects. The permeability, RoCA, and resistance to aggressive environment and other external attacks of POFA are described in subsequent subsections.

### 4.1. Permeability

**Figure 7:**
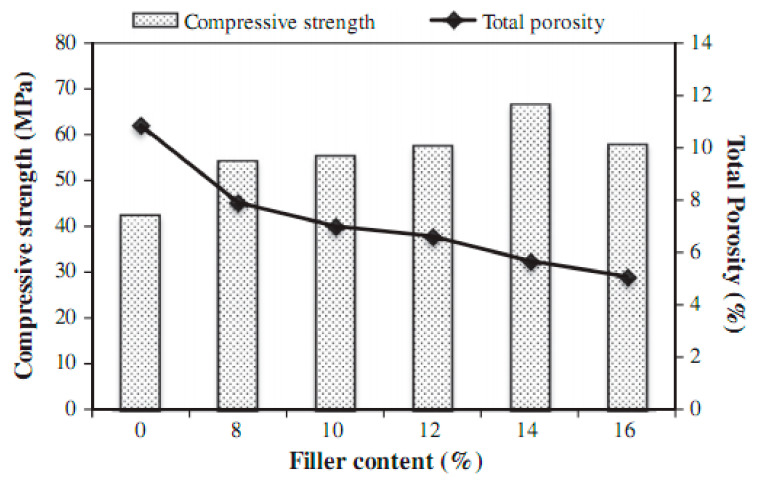
Water permeability test of concrete [109]. Reprinted with permission from Elsevier [109].

Permeability describes the rate of infiltration of a liquid (water) and other aggressive particles into the POFA concrete and originates carbonation, chloride-ion attack, and corrosion [107,108]. Reducing permeability can help to decrease the entrance of acids into the POFA matrix; this subsequently improves the concrete resistance to acid attack [109]. Figure 7 shows the correlation between porosity (concrete with more porosity tends to be more permeable) and compressive strength and filler content. Governing the permeability of concrete is significant as the ingress of atmospheric ions through the construction materials is primarily at fault for structural degradation [75]. The water permeability of concrete with POFA relies mainly on the content volume and the grade of fineness of POFA. Reports indicated that, with an increase in ground POFA, the permeability of water decreases with increasing age, due to the creation of extra gel during the pozzolanic reaction of ash [28,32,45,58,75]. For example, it was found that in concretes containing ground POFA in cement at the 20% and 40% levels the penetrability of water of POFA-based concrete was less than that of the reference samples of 28 and 90 days [40]. Concrete permeability of mortar fabricated using 55% POFA quickly improved and was greater than that of normal concrete [110,111]. This result is possibly due to the high w/b ratio and low OPC content of concrete prepared with 55% ground POFA [111,112]; mortar comprising 20% POFA, however, demonstrated the lowest rate of water penetrability in comparison to other dust contents. Furthermore, all the concretes with ultra-high-strengths consisting of POFA resulted in 50% lesser permeability of water and other aggressive elements compared to OPC concrete [45]. In general, POFA improved the impenetrability of concretes/mortars by means of lessening the porosity as well as pore refinement.

Water absorption (WA) is usually calculated using Equation (1).

(1) $$WA\;\left(\%\right)=(\frac{w_{1}-w_{2}}{w_{2}})\times \%$$

where

*w*_1_ = weight of saturated specimen.
*w*_2_ = weight of oven-dry specimen.

The water permeability coefficient of POFA concrete can be calculated using Equation (2).

(2) $$K_{p}=\frac{d^{2}v}{2ht},\;v=\frac{m}{Ad\rho }$$

where

*K_p_* = coefficient of water permeability (m/s).
*d* = depth of water penetration into concrete (m).
*v* = porosity of concrete.
*h* = hydraulic head of water (m).
*ρ* = water density (1000 kg/m^3^).
*t* = time under pressure (s).
*m* = gain in weigh (g).
*A* = area of sample (mm^2^).

Void content,

(3) $$\%=[1-(\frac{A-B}{\rho_{w}\;Vol})]\times 100\%$$

where

*A* = dried-weight (g).
*B* = saturated-weight (g).
*Vol* = sample volume (cm^3^).
*ρ_w_* = water density at 21°C (kg/cm^3^).

Numerous researchers have previously applied the falling-head experiment to obtain the water permeability coefficient of concrete Figure 7. The coefficient of water permeability is computed using Darcy’s Equation (4):

(4) $$K=\frac{A_{tube}\times L}{A\times L}\times \ln\left[\frac{h_{1}}{h_{2}}\right]$$

where

*K* = coefficient of water permeability, (mm/s).
*A_tube_* and *A* are the areas of the tube and sample, (mm^2^).
*L* = sample length, (mm).
*t* = time needed for water to drop from level *h*_1_ to level *h*_2_, (s).

### 4.2. Rate of Capillary Absorption

The rate of capillary absorption [113] is related to the porosity of a pore structure where the water ingress rate and any fluids are extensively governed by absorption as a result of the capillary rise of unsaturated concrete [114]. RoCA is one the most significant features of a construction material, as it controls the movement of moisture through the material and expresses the RoCA of water down to capillary powers for construction materials. The RoCA of POFA concrete increases over time, and the RoCA of other materials increases along with their RoCA contents, except when the RoCA content is 15%, which leads to slightly less than that of POFA concrete, which also contains 100% normal-CA [110,114,115]. The absorption of water in concrete improved with an increase in POFA content, thanks to the fact that a high unground POFA content could contribute to forming a more porous concrete matrix [41,75]. This result shows that concrete consisting high amounts of unground POFA content is inclined to capture more water due to a higher porosity [116,117]. However, the absorption of water in concrete, possibly condensed from the addition of ground POFA because of its PA and acceptable micro-filling ability, contributes to pore refinement. It has been reported that concretes containing between 10% and 50% POFA with 100 mm-diameter samples revealed water absorption rates of 6.64% and 8.90%, correspondingly, using the initial-surface-absorption-test, whereas the absorption value of the control was 6.20% [116,117,118]. These results showed that the lowest rate of flow of absorption for the entire ratios was observed for the samples encompassing 10%-POFA [43,49,117,119]. Adding foam improved the absorption of water in POFA concrete as its usage prompted extra pores and pored connectivity. This phenomenon contributes to an upsurge in water absorption and in the sorptivity of concrete, leading to a lessening in compressive strength [109]. FC can be categorized as regular quality, or classified as poor, moderate, and good when the water absorption is at a low rate of 3%, limited to 3%, 5%, and larger than 5%, respectively [120]. However, the coefficient of water absorption depends on the temperature that is often adopted, but this is obviously inexact. This reliance should be deliberated by many researchers and specialists who use the coefficient of water absorption as a key to describe a building material [121].

### 4.3. Chloride Penetration

**Figure 8:**
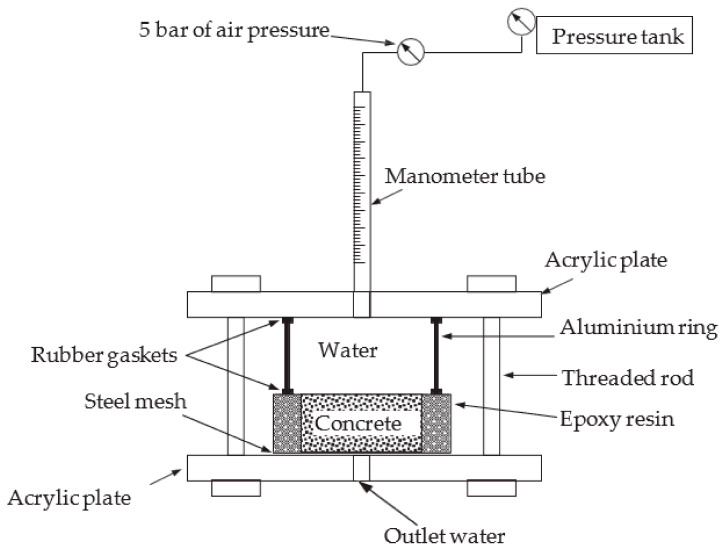
Water permeability test of concrete [40]. Reprinted with permission from Elsevier [40].

Corrosion is one of the main problems in RCs utilized in infrastructure; for instance, harbor and offshore constructions, bridges, and pavement [122]. Chloride penetration (CP) is measured according to ASTM C 1202 [86]. The corrosion of reinforcement bars in RC is believed to be prompted by CP. The entrance of chloride salts into a concrete matrix is augmented by the cyclical exposure to seawater wetting and air drying [123]. However, ASTM C 1202 recommends maintaining particular standards, which are detailed in Table 1, for quality control and acceptable testing applications [86]. Several specifications outline how the charges are introduced, as well as the recommended values. In several SCMs, such as fly ash and slag, these benchmarks may not be valid until 56 days or at a later curing age; thus, testing for overall chloride penetration at 28 days of curing poses a problem [49,123,124,125]. Figure 8 displays the results of the rapid chloride penetration test (RCPT) [40]. The outcomes of the RCPT for OPC and POFA-based concrete were done previously [124,126] (Table 1). Applying 60 V for a period of 6 h at either end of OPC/POFA-based concrete samples gives RCPT values of 731.7 coulombs and 276 coulombs, respectively. Several experimental investigations [127,128] found that ground POFA may possibly be of use as a partial substitution for PC to make a concrete with a significant resistance to CP. Reportedly, concrete comprising 60%, 40%, and 20% ultra-fine POFA revealed an 84% reduction, using the rapid CP test, in comparison with the reference mix when 60% POFA was used [56]. This shows that the use of POFA contributes to a small coefficient of chloride dispersion and decreases the condensation depth outline of the liberated chloride compared to the cement [42]. The coefficient of chloride dispersion was absolutely interrelated with bigger pore sizes, such as 25 mm in thickness [64,93,129]. The upsurge in the fineness and level of POFA in concrete results in a decrease in liberated chloride and dispersion coefficient of chloride [125,129]. Through the RCPT, as detailed in ASTM C 1202, concrete consisting in 20% POFA showed that the average charge that passed was 276.3 C while that of the reference concrete was 731.7 C [124]. The depth of CP ions into the POFA concrete was less compared with that of the OPC concrete. Accordingly, POFA reduced concrete permeability, thus improving resistance to CP. It was reported that a passing charge may be condensed substantially with 20% POFA in comparison with normal-weight concrete [40]. Meanwhile, 10% silica fume replacement could decrease the introduced charge by almost 60% in comparison with OPC-based concrete [125]. The pozzolanic reaction ameliorates the interaction between the cement and aggregates matrix, leading to the formation of dense and resistant concrete [45,130]. In another study, researchers executed a RCPT test using POFA from three dissimilar PO mills, which were designated as KTPOFA, ALPOFA, and CAPOFA [124]. The investigation revealed that when 60 V was applied for 6 h at either end of a sample, this resulted in RCPT values of 731.7 C. Additionally, considering ALPOFA-, KTPOFA-, and CAPOFA-based concrete, typical passing of charges were 380.25, 463.25, and 276.3 C, respectively [45]. A chloride migration experiment was accomplished in line with the AASHTO standard [131], and the chloride penetration rate (CPR) could be computed using Equation (5),

(5) $$C_{PR}=\frac{X_{d}}{t\times U}$$

where

C_PR_ = CPR, (m/(v.hr)).
*U* = absolute value of the used voltage, (V).
*t* = time of test (h).
*X_d_* = penetration depths, (m).

### 4.4. Resistance to Sulfate Attack

The choice of materials and the proportioning of concrete in susceptible zones should be precisely determined to withstand external attack [122,132,133]. This phenomenon is known as sulfate attack and it can be determined in accordance with ASTM C 1012 [134]. External sulfate attack occurs when encompassing water dissolves sulfates, and penetrates concrete [28,119,124]. External sulfate attack encompasses thaumasite formation, calcium aluminate reactions, and crystallization of sulfate salt. Interior sulfate attack initiates when the sulfate basis is associated in the mortar during mixing [110,124]. Sulfates react with the hydrated calcium aluminates to create calcium sulfoaluminates, which is improved by nearly 227% compared to liberated calcium hydroxides and authentic aluminates in the paste in the production of calcium sulfate [75]. Such a reaction contributes to the production of thaumasite and gypsum, resulting in harmful effects, such as cracking, spalling, strength loss, and softening, as well as additional types of concrete destruction. Their amplification causes cracks creation followed by the deterioration of concrete [75,135]. Any change in concrete mixtures cannot diminish the performance of the material because concrete is commonly exposed to severe conditions, such as sulfate attacks. POFA possesses many advantages in concrete construction, which enhance concrete properties in order to resist sulfate attacks [28,136]. POFA has a better resistance to sulfate attacks (RSA) than OPC because of its alternative low-toxicity coating material [45,119,137]. Generally, the extension of concrete attributed to sulfate attack reduces with an increase in POFA content [45,136]. It is noted that fineness influences the sulfate resistance of concrete; that is, the coarser the POFA, the greater the enlargement of concrete caused by attack of sulfate. Furthermore, examples encompassing ultrafine POFA and ground steel slag to 5% MgSO_4_ and 5% Na_2_SO_4_ for 182 days. Thanks to active interactions, CaSO_4_ depositions cause a decrease in MgSO_4_ with an upsurge in ground slag, resulting in a reduction of strength and weight of the samples, contrary to what occurs in samples exposed to Na_2_SO_4_ [75]. This finding may be due to that the fact that microstructural constancy of samples was observed in the samples exposed to MgSO_4_ compared to Na_2_SO_4_. The addition of GGBS and metakaolin (10% to 15%) to POFA admixtures can also increase the RSA of concrete and significantly reduce its alkali–silica reactivity, as shown in the following equations [138].

Acid-based reaction:

H_0.38_ SiO_2.19_ + 0.38NaOH → Na_0.38_ SiO_2.19_ + 0.38H_2_O

Siloxane bridge attack and silica disintegration:

$${\mathrm{Na}}_{0.38}\;{\mathrm{SiO}}_{2.19}+1.62\mathrm{NaOH}\to 2{\mathrm{Na}}^{2+}-H_{2}\mathrm{Si}O_{4}^{2+}$$

Sulfate attack reduces concrete durability by altering the natural chemical composition of the adhesive and the mechanical characteristics of the concrete [128]. The hydrated calcium aluminate + sulfate ion and the calcium-hydroxide modules of water + hardened OPC paste = calcium sulfo-aluminate hydrate is expressed (I and II) as:

12H + 2CH + C_3_A.Cs.H_18_ +2s = C_3_A.3Cs.H_32_ (I)

11H + 2CH + C_3_A.CH.H_18_ +3s = C_3_A._3_Cs.H_32_ (II)

The hydrated calcium aluminate + sulfate ion or the calcium-hydroxide compositions of water + mechanical cement paste = gypsum are expressed (III and IV) as:

2H_2_O + Ca(OH)_2_ + Na_2_SO_4_ = CaSO_4_·2HO + 2NaOH (III)

2H_2_O + Ca(OH)_2_ + Mg_S_O_4_ = CaSO_4_·2H_2_O + Mg(OH)_2_ (IV)

It is also shown that the addition of finer POFA diminishes the overall content of calcium hydroxide and the expansion scale of the concrete bars, but it also promotes as plaster, contributing to a decrease in holes among the hydration products and aggregates, tending to produce a heavier concrete [46,75,92]. Meanwhile, the use of 10-μm POFA and recycled aggregate exhibited a reduction in the strength and expansion levels compared to the reference mix [110]. This is because when cement is a partially-substituted, the volumes of C_3_A and Ca(OH)_2_ are condensed in a solidified concrete, hence decreasing the fabrication of ettringite and gypsum re-crystallization.

### 4.5. Resistance to Acid Attack

**Figure 9:**
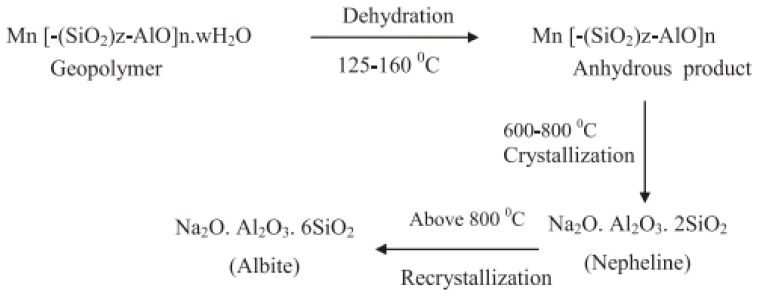
Influence of POFA on the concrete resistance to sulfate [40]. Reprinted with permission from Elsevier [40].

Concrete is known to be very alkaline in nature and extremely susceptible to acid attack [122,132]. Acids attack concrete by melting both unhydrated and hydrated cement composites and calcareous aggregates [33,92]. Resistance to acid attack (RAA) in concrete can be measured according to ASTM C 106 [128,139]. Degradation can occur if the concrete is unprotected to destructive sulfuric acid environments [75]. One vital durability issues for all concrete constructions is RAA, in particular, in industrial zones owing to acid rains that cause sulfuric acid [37,128]. Sulfuric acid may be present in ground water and in chemical waste [92,140]. Sulfate attack is less calamitous than sulfuric acid attack as a consequence of the suspension impact by sulfate ions plus the attack by the hydrogen ions [140]. Investigations have revealed that the addition of 20% pulverized POFA enhanced paste against acid attack [45,58,61,126,141] Figure 9. These results indicated superior immovability of POFA due to the greater rates of finer particles and silica content. It was reported that both concretes containing either 30% ground POFA or OPC concrete, were submerged in a 5% hydrochloric acid solution; it was observed that the loss of mass of POFA-based concrete after 2.5 months was low compared to reference samples [39,140]. Thus, the developed microstructure of the concrete was denser with a decrease in porosity [117,139]. This phenomenon was attributed to a condensed diffusion of acid smelting inside the internal texture of the concrete [128]. POFA concrete exhibited a stronger microstructure than that of normal concrete after exposure to acid solution due to the contribution of the low lime content and the PA POFA concrete [139,140]. The volume of porous Ca(OH)_2_ was low due to the small amount of lime content [117]. The extra C–S–H gel from the reaction of pozzolanic ash can was made at the outlay of Ca(OH)_2_. Reports indicated that the most susceptible element of hydrated cement is Ca(OH)_2_, when acid can still attack the C–S–H gel [139]. Concrete elements in manufacturing areas are also susceptible to worsening weather, which is attributed to acid rain in which sulfuric acid is a key material [142]. Studies have stated that samples of concrete encompassing pulverized fuel ash (PFA) and POFA, engrossed in a 2% sulfuric acid solution for 1.5 years, showed only an 8% loss in the mass of the PFA and POFA concretes and a 20% loss in the reference concrete according to optical examinations [140,142]. It was also seen that, when the samples strength was assessed after acid attack, simple worsening and a 35% strength loss in the ash-based concrete was found, even though the loss of the OPC-based concrete was only 68% [139]. In general, concrete corrosion by sulfuric acid can be categorized by the following reactions (V and VI), as stated by Ariffin et al. [142]:

Ca(OH)_2_ + H_2_SO_4_ = CaSO_4_·2H_2_O (V)

3(CaSO_4_·2H_2_O) + 3CaO·Al_2_O_3_·12H_2_O + 14H_2_O = 3CaO·Al_2_O_3_·3CaSO_4_·32H_2_O (VI)

CaSiO_2_·2H_2_O + H_2_SO_4_ = CaSO_4_ + Si(OH)_4_ + H_2_O (VII)

### 4.6. Resistance to Alkali–Silica Reaction

The alkali–silica reaction (ASR) is more generally recognized as a cancer of concrete, and is a bulge activation that arises with age in concrete with extremely alkaline-paste and volatile amorphous silica that presents in various popular aggregates with a specified adequate moistness [122]. ASR is known as a concrete durability problems, where particular types of silica found in aggregates interact in high alkaline pore solutions in concrete to create a reaction artifact that increases in the presence of moisture and causes venomous cracks patterns in concrete textiles [132,143,144,145]. ASR can be assessed according to ASTM C 1260 [146]. Substituting part of a cement with PCR is effective in controlling the extension of ASR within concrete and mortar [75,147]. Preceding investigations have stated that the main mechanism of how PCR diminishes ASR expansion is by reducing the hydroxyl ion (Off) in the pore-solution [39]. Other mechanisms of ASR suppression occur through alkali binding, limiting permeability, improving pozzolanic reactivity, and reducing the dissolution of silica [59,126,140] The effectiveness of PCR in reducing ASR expansion depends on quite a number of factors, such as reactivity of aggregate, alkali content of OPC, and the fineness of PCR [147]. Research on POFA has revealed that POFA can mitigate ASR damage since a large number of substitutions is required to control the effects of ASR [39]. However, this significantly reduces the strength of the developed mortar [41,49,135]. Another study on POFA showed that the effectiveness of POFA in controlling the ASR reaction could be improved by reducing POFA to an ultrafine size [146]. For instance, concrete containing 10% ground POFA showed a nearly 25% reduction after 12 days of exposure [39,53]. A significant decrease in expansion for 50% POFA was also found [148]. Thus far, POFA has exhibited a good potential for suppressing the expansion associated with ASR reactions in concrete [41,149]. The high alkali content present in POFA was sufficient to drop the extension owing to the reaction of ASR [39,61,124,129]. Since the particles of POFA react so quickly with the alkalis found in OPC, as a result of their natural reactiveness, there were many quite tiny unreacted alkalis for subsequent interaction with reactive aggregates [39,150,151]. In addition, rice husk, calcined clay, and metakaolin, as well as calcined shale, can be well preserved using an oven and subsequently PFA with a finer powder is produced, from which a majority of common natural pozzolans originate [65,138]. Incorporating these pozzolanic materials to a replacement level of 15% to 35% in cement improves resistance to sulfate attack, permeability, in addition to controlling the alkali–silica reactivity [39,150]. However, the mechanism of how ultrafine POFA controls the ASR has not yet been studied. Despite having a higher alkali content than cement, POFA is still effective in suppressing ASR expansion; therefore, further research is necessary to reach a solid conclusion on the influence of ASR in POFA-based concrete.

### 4.7. Resistance to Carbonation

The carbonation of concrete, among other factors, is one of the primary reasons while reinforcement bars corrode [122]. It is a process of carbon dioxide penetrating from air into a concrete matrix where it reacts with Ca(OH)_2_ to shape calcium carbonates [45]. Carbonation frequently occur in concrete as a result of extant calcium tolerance stages that are punished by CO_2_ in the atmosphere and are transformed into calcium carbonate [132]. The paste encompasses 25–50% Ca(OH)_2_, indicating that the pH of fresh concrete paste has a minimum of 12.5 [93]. Carbon dioxide can infiltrate concrete surfaces and interact with the compositions of alkaline, principally the Ca(OH)_2_ of mixtures. This carbonation activation possibly decreases the pH value to less than 9 in the pore solution [75]. It was noted that when the pore sol alkalinity is missing, the protection of the steel bars, in contrast to corrosion, is at risk [46,75]. The characteristics of concrete, for instance shrinkage, permeability, strength, and resistance to physical/chemical attacks, are mostly influenced by carbonation [152]. A certain amount of the released CO_2_ is re-ingested from the air during the carbonation process of concrete [114,152]. Investigations on the resistance of carbonation in concrete containing only POFA and OPC, the results showed that the increase in hydration caused in an upsurge in expended Ca(OH)_2_ in the mass of concrete, and hence could contribute to an improvement in carbonation of mortar [152]. Nevertheless, the upsurge in hydration composition with the padding influence made a heavier and stronger matrix to withstand the ingression of carbon dioxide, and therefore depressed carbonation [45,153]. This finding was evidently affected by the fineness and the higher PA of POFA [75,129,154,155,156]. POFA can possibly be used as a decent pozzolan to partially replaced the OPC in producing paste yielding a very high strength and low carbonation depth, and thereby concrete can be for indoor use [75,154]. This will lessen the charge of concrete and have a positive outcome for ecological issues and decrease the number of landfill areas needed for the dumping of waste ash [75,135].

### 4.8. Acoustic Insulation Resistance

The AIR of POFA concrete can be evaluated according to ASTM C 423 [157]. Thin foamed concrete (GFC) specimens (20 mm to 25 mm) display a remarkable rate of acoustic absorption (*α* = 0.7 to 1.0) in a small-frequency zone of 40 to 150 Hz [158], as a result of variations in their pore diameters, tortuosity, and porosity upon supplementation with slag [149]. However, the increase in the thickness of the GFC samples can also significantly improve their sound absorption in the low-frequency zone by increasing the volume of materials over which the sound waves need to be transmitted [132,149,157]. However, a thickness increase shows an insignificant effect at higher frequencies. A sound with a higher wavelength (lower frequency) shows a higher sensitivity to the material thickness of POFA concrete specimens [118]. The increase in the foam dosage, from 5% to 10%, creates a material that cannot effectively absorb low-frequency sounds but can effectively absorb medium-frequency (600 Hz to 1000 Hz) sounds; in contrast, a standard density PC concrete frequently has a coefficient of acoustic absorption of *α* < 0.1 at a frequency of 100 Hz to 2000 Hz [159]. GFC also shows excellent acoustic absorption characteristics. Thin GFC samples demonstrated less absorption of sound within the average to higher-frequency zones compared to permeable concrete, which has a regular coefficient of acoustic absorption of *α* > 0.5 in these zones [158]. Using slags to substitute 30% FA in concrete does not meaningfully alter the absorption of sound in the small-frequency zone, but increases the absorption of sound at maximum frequencies, specifically from 800 Hz to 1600 Hz [158]. Any additional increase in the thickness of GFC samples will increase their AIR. The normal incidence acoustic absorption coefficient (*α*) is computed as:

(6) $$\alpha =1-R^{2},\;R=\frac{e^{jkd_{1}}-Pe^{jkd_{2}}}{Pe^{-jkd_{2}}-e^{-jkd_{1}}}$$

where

*R* = reflection coefficient.
*j* = wave number.
*d*_1_ and *d*_2_ = distances between the sample surface and the far and close active microphones, accordingly.
*P* = sound pressure ratio at the dual active microphone rooms.

### 4.9. Thermal Conductivity

Thermal conductivity is an important property that can improve the energy efficiency and environmental sustainability of modern buildings [160]. Thermal conductivity can be tested according to ASTM C 1363 [161]. At high temperatures, knowledge of the performance of concrete is primarily of great importance for designers of RC structures [122]. The thermal conductivity of a device is the amount of temperature transferred by a depth in the vertical axis to the surface area, attributable to the temperature gradient under specific conditions [162]. As a new construction material, POFA concrete has a favorable thermal insulation and thermal conductivity because of its light weight [158]. The bowed surface of POFA has several small pores with diameters limited to 16–24 μm and, therefore, the thermal insulation property of POFA from the air stuck inside POFA can possibly be exploited [148]. In addition, utilizing the foam to have more air voids in OPSC could possibly improve the thermal insulation characteristics of POFA [160]. The 28 day-thermal conductivity of FC comprising 20% POFA was investigated by Liu et al. [163]. In their findings, it was found that overall thermal conductivity decreases with an increase in bubbles in concrete when compared with the normal bricks and blocks utilized in walls; the thermal conductivity of foamed POFA concrete recorded 22% and 48% less, respectively. It was also reported that samples containing 10% and 20% POFA recorded thermal conductivity values of 0.73 and 0.68 W/mk, correspondingly, whereas the value of the reference concrete was 0.65 W/mk. Moreover, POFA concrete increased in strength when subjected to temperatures in the range of 500 °C [37,59]. Expanding POFA content alters the temperature of the most extreme strength, and examples lose strength when the temperature is expanded beyond 500 °C. Table 2 displays the thermal conductivity values in POFA concrete applications reported by a number of researchers.

### 4.10. Fire Resistance

**Figure 10:**
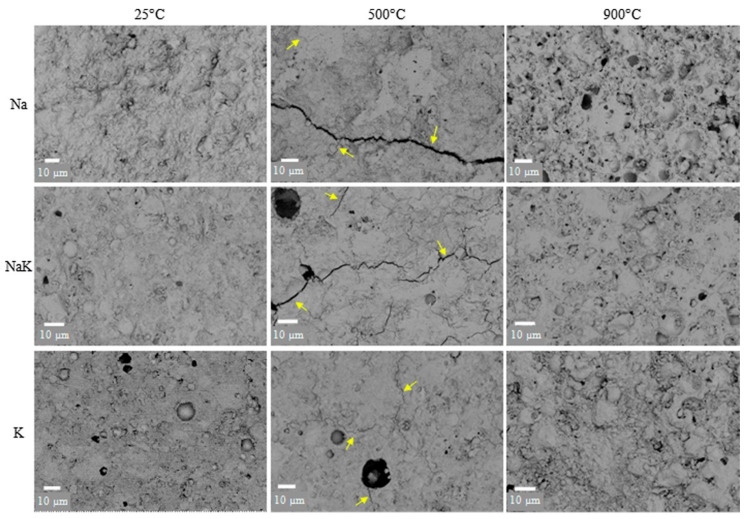
Formulation of micro-cracks in geopolymers upon the exposure to high temperature [169]. Reprinted with permission from Elsevier [169].

Construction materials with advanced properties, such as superior fire resistance (ASTM E 2748), have recently been in demand [108]. As the primary contributor to the fire resistance of concrete, water accumulated inside the geopolymer structure is released during the heating process to reduce the heat temperature and to form a porous microstructure [122]. Fire resistance is a significant problem since concrete should have the ability to reserve its mechanical behaviors for a given lifespan; this is recognized as the rate of fire [132]. Fire is one of the most destructive influences that can expose concrete to extreme temperatures, and are consequently among the greatest risks that buildings are exposed to [45,54,59,124]. The spalling of concrete during fire will cause a speedy layer-by-layer disintegration of concrete cover and finally degenerating to the expose the main reinforcements within the concrete to the fire [41]. Recent studies found that making concrete with PCR with SCMs is one of the most effective methods in enhancing the fire resistance of durable concrete [45,54,124,126,164]. The fire resistance test reveals that this ash-based porous concrete can perform remarkably well in every test [132]. While the examined material showed changes in color from black to grey, it was still able to show a significant fire resistance [75]. The behavior of a POFA-based concrete in the early phases of a fire was also investigated by performing tests in line with BS 476 Part 6. The type of cement, aggregates, moisture content, and permeability influence the fire resistance capacity of OPC concrete [71,102]. It is revealed that a sensible selection of materials could permit OPC concrete strength to be sustained despite damages at heats equal to 600 °C being inflicted [165]. When samples with 20% POFA concrete were exposed to 800 °C, the resulting damage was lower in comparison with the OPC at temperatures less than 500 °C [93,166]. This can be a contribution of the lower content of Ca(OH)_2_ in the POFA mortar [37,166]. However, the addition of ash-based cement paste can increase the fire resistance properties and reduces the dispersion of fire [45,167,168]. This agro-based cement leads to lower CO_2_ emissions than OPC [27,45]. As stated, during a fire Figure 10 [169], geopolymer concretes have additional micro-structure variations that are attributable to the difference in coefficient of thermal expansion of aggregates and geopolymer paste [169]. Several events, such as the absorption of water by N–A–S–H gel, the creation of anhydrous products, the crystallization of stable anhydrous phases, and sintering, can all contribute to the devastation of concrete.

## 5. Beneficial Use of POFA

### 5.1. Ecological Benefits of POFA

In industrial sectors, cement consumes 12–15% of the allocated energy [170], which entails the combustion of fossil materials, such as fuels, petroleum coke, as well as coal, to maintain 1450-°C oven temperatures [171]. As a result, nearly 0.97 tons of carbon (IV) oxide (CO_2_) is generated for every ton of clinker production [45]. This produces approximately 7% of CO_2_ gas emissions. Cement manufacturing was predicted to have increased by 100% from 2008 to 2020 [148]. Referring to the cement production rate in 2010, it was predicted that demand may reach 200% by 2050 [172]. Moreover, for the production of 1 ton of cement, the energy consumption exceeds 1700 MJ/ton clinker [173] with 1.5 tons of natural materials. From a previous report, about 5% of total energy is sourced from the cement industry [174] and it has become the second largest CO_2_ gas emissions contributor worldwide [175].

There are several environmental issues, and global warming is one of them, which is partially induced by industrial and agricultural solid waste materials [25]. A previous study [176] stated that untreated POFA disposal in open landfills may threaten human life. As the demand for new buildings for development is increasing, CO_2_ generation will damage the atmosphere [43,177,178]. To minimize this impact, researchers have suggested how to mitigate this risk by applying these agricultural wastes in cement mortar [37] or in the construction industry [40]. The high silica content of POFA can be used as a cementitious material to produce sustainable construction materials [25]. By utilizing them in construction as a “second life”, POFA, a pozzolanic material, is able to reduce environmental problems by reducing the burden on landfills and lowering CO_2_ emissions [28].

As there is an increasing demand for energy resources and new renewable resources are urgently needed; non-renewable resources from petrochemicals and natural gases should be consumed less, as they may contribute to global warming [75]. POFA concrete is termed as green or sustainable concrete due to it using less energy in its production compared to cement. In fact, it helps to minimize CO_2_ gas emissions by 5 to 8% of the level generated by cement production [179]. The untreated wastes from the palm oil industry may cause serious ecological problems [180]. It has also been reported that has been an increasing trend in palm oil product demand, consequently increasing waste materials [176]. Other than property enhancement, incorporating POFA into concrete also reduces the concrete production costs and improves environmental conditions [181].

Concrete matrix formed from biomass products should cause less environmental damage compared to traditional chemical materials. POFA, as cement replacement in concrete, may lead to better solid waste management [182]. As POFA may minimize greenhouse gas emissions [73], it may also significantly improve environmental issues in the long term [25,90]; POFA also enhances concrete characteristics, such as mechanical properties and durability, in addition to chloride and sulfate resistance. Generally, most research is concentrated on waste utilization in concrete matrix, aiming to solve environmental problems while enhancing concrete properties. Air pollution can be minimized using POFA; however, to date, no research has mentioned the influence of POFA on water and soil pollution, which should be addressed by current research trends.

### 5.2. Nano POFA

Nanotechnology, a current trend, has found applications in various industries [23] and has been applied in concrete due to its unique characteristics [183]. Nanoparticles are sized less than 100 nm [184]. The incorporation of nano-materials into a concrete matrix enhances its characteristics, such as mechanical and durability properties; these materials are referred to as nano SiO_2_, silica fume, nano-clay, and nano fly ash, in addition to carbon nano-tubes [185,186]. Nano POFA has also been studied and found to improve concrete properties [42,71] by reducing the amount of calcium hydroxide. Cement hydration and microstructure of cement paste with nano POFA as a pozzolanic material has been investigated [183]. POFA is considered a pozzolanic material with a substantial quantity of SiO_2_ [187]. A more green concrete with improved compressive strength can be produced by incorporating a large volume of nano POFA [188]. Several studies showed that initial particle sizes and the porosity of POFA are not beneficial to concrete microstructure [28,41,176] and it has been suggested to pulverize POFA for more fine particles [54,97,155], thus increasing the rate of chemical reactions with a higher surface area and produce higher compressive strengths [28].

Nano particles can be used to represent the ground POFA in order to increase reactivity with other constituents and enhance concrete properties [54,155]; 7.4 µm POFA with a cement replacement level of 10% was found to be the same as the control specimen [28]. A higher degree fineness of POFA can be used as a replacement for type I Portland cement with as much as 30% weight of binder [42]. An 80% replacement level of nano-POFA in cement with particle sizes lesser than 1 µm was able to produce a high compressive strength and a high quality concrete [56]. In a fresh state, ultrafine POFA also increased concrete workability and delayed concrete setting time when there was a higher content of POFA [56]. UPOFA is classified as a class F pozzolan with a higher content of silica, while GPOFA is classified as class C pozzolan [189]. However, to date, concrete composites or a matrix with all nano particle materials have yet to be investigated, and it is believed that using nano materials may have better properties and should be considered in current research planning.

### 5.3. Nano Silica with POFA

Spherical particles of nano silica, sized 1–50 mm, can enhance fresh and hardened concrete properties [190]. In term of compressive strength and dry shrinkage, nano silica POFA concrete with 30% cement replacement was studied, and a 15% strength enhancement and a reduction of shrinkage was found compared to control specimens [190]. It was revealed that a compressive strength increase and a reduction in water absorption was found for unground POFA cement mortars using nano silica [43]. It was reported that compressive strength was increased in the first day of curing with nano silica due to larger surface areas with even C–S–H distribution; thus causing a greater extent of hydration reactions [191]. It was reported that nano silica is the one of the most common nano particle materials in concrete matrix and it has been investigated [192] using various of quantities [193,194,195,196]. Small amounts of nano silica, between 1 to 3%, were able to improve concrete properties, particularly durability, compressive strength, and composite microstructures. The high surface areas of nano silica provide high reaction activities and thus accelerate C_3_S dissolution [197]. In term of physical properties, calcium hydroxide (CH) usage and the formation of C–S–H clusters of nano silica were able to improve the pozzolanic properties [196]. As POFA possesses a high content of silica, ranging from 50 to 70% of total weight, it is a good pozzolan and it was not necessary to add more silica to the design mixes [181]. The fast CH consumption was investigated for early concrete ages to determine the high reaction rate of nano SiO_2_ in terms of the properties of mortar paste and concrete mixtures. Nano silica in POFA concrete also showed a positive influence on concrete shrinkage beyond the microstructure [190]. In general, more information is needed and conducting further experiments with nano silica POFA concrete under various conditions is needed.

## 6. Conclusions

The majority of preceding research has concentrated on the specific characteristics of POFA concrete, for instance its high compressive strength and PA, rather than on the morphological micro-structure characteristics. This research also ignored some components, for instance artificial and natural fibers, as well as alkaline activator solutions, and how they affect the strength of POFA concrete matrix. For example, a binder paste that contains a small amount of carbon nano-fiber reveals great stability and sensitivity in POFA concrete. The subsequent conclusions can be noted on the basis of the observations from this study review regarding POFA in mortar/concrete. The inclusion of POFA as a PCR in concrete mixes will ameliorate the challenges due to agro-based waste disposal and subsequent environmental issues, reduce the ecological vulnerabilities assumed by ordinary Portland cement plants, and reduce to a considerable extent the greenhouse gases (CO_2_, methane, CO, etc.) emissions in the air while reducing the overall cost of cement usage. Considering the fresh and hardened characteristics of POFA their use for making concrete is pivotal. Based on this review, the fineness of POFA is an important property in concrete; however, the great fineness of POFA enhances its capability for micro-filing and PA, thereby leading to enhanced concrete mechanical characteristics and durability properties. POFA-based composites presented a similar and, from time to time, superior performance than normal concrete in withstanding carbonation, sulfate resistance, as well as acid attack. Further investigation is suggested to determine the overall influences of POFA on various characteristics of concrete, in addition to durability properties, and therefore inspire the inclusion of POFA in producing concrete. These are highly imperative for continued incorporation of POFA in self-compacting concretes (SCC). POFA has been used as a potential binder (as a PCR) up to a particular substitution level of OPC in which is safe from any negative influence on the strength or/and durability of concrete. In conclusion of this review study and to address the favorable influences of POFA on some concrete properties and durability problems, which make POFA an alternative PCR in concrete, several research investigations are recommended for the future:

- To investigate the effects of POFA on the slump loss, plastic shrinkage and the air content of concrete while examining the influences of POFA on rheological characteristics, such as plastic viscosity and yield stress of concrete;
- To examine the effect of POFA on the bond, tensile, fatigue, impact, shear, and flexural strengths of concrete; and to study the feasibility of using POFA concrete in resisting aggressive environments;
- Determination of the effects of POFA on the autogenous, creep, water absorption, drying shrinkage, thermal resistivity, oxygen penetration, as well as diffusion of aggressive ions in concrete;
- Evaluating the durability performance of POFA concrete with regards to de-icing, salt-scaling, freeze and thaw, abrasion, and corrosion resistance.

General investigations into the properties of high fineness POFA with the aim of enhancing the micro-structure, which can result in a dense extremely impervious concrete matrix is highly recommended; in particular to investigate the performance of POFA materials incorporated in ultra-high-strength and self-compacting concretes while enhancing their mechanical and durability properties using fibers.

## Figures and Tables

**Table 1:** Chloride ion penetrability depending on the extent of passing charge [86].

Penetration of Chloride Ion	Passage of Charge (Coulombs)
Negligible	<100
Very low	100 to 1000
Low	1000 to 2000
Moderate	2000 to 4000
High	>4000

**Table 2:** Values of thermal conductivity.

Ref.	[71]	[160]	[65]	[148]	[159]	[37,119]	[65]	[65]	[75]
Thermal conductivity (W/m.k)	0.19	0.40	0.74	0.57	0.74	0.69	0.65	0.74	0.67

## Data Availability

Data sharing not applicable.

## Abbreviations

AIR	Acoustic insulation resistance
ASR	Alkali–silica reaction
CAPOFA	Calcium salt palm oil fuel ash
FA	Fly ash
GFC	Foamed concrete
OPC	Ordinary Portland cement
POFA	Palm oil fuel ash
PCR	Partial cement replacement
PFA	Pulverized fuel ash
RCPT	Rapid chloride penetration test
RoCA	Rate of capillary absorption
RC	Reinforced concrete
